# Impact of extruded flaxseed meal supplemented diet on growth performance, oxidative stability and quality of broiler meat and meat products

**DOI:** 10.1186/1476-511X-12-13

**Published:** 2013-02-08

**Authors:** Faqir Muhammad Anjum, Muhammad Faizan Haider, Muhammad Issa Khan, Muhammad Sohaib, Muhammad Sajid Arshad

**Affiliations:** 1 National Institute of Food Science and Technology, University of Agriculture, Faisalabad, Pakistan

**Keywords:** Broiler meat, Extrusion, Flaxseed, Nuggets, PUFA, Lipid stability

## Abstract

This study was intended to explore the effect of extruded flaxseed meal supplemented diet on broiler growth performance, oxidative stability and organoleptic characteristics of broiler meat and meat products. 120 (day old) broiler chicks were randomly allotted to 12 experimental groups and fed on diets containing extruded flaxseed meal at 0, 5, 10 and 15%. The supplementation of extruded flaxseed in the diet decreases the body weight gain, feed intake and increased feed conversion ratio (FCR) values of broilers. The antioxidant enzymes were strongly influenced by different levels of extruded flaxseed supplementation among treatments. The TBARS assay revealed that maximum malondialdehyde were produced in T_3_ containing highest extruded flaxseed level (15%) and minimum malondialdehyde were produced in T_0_ treatment having no extruded flaxseed. The TBARS values ranged from 0.850-2.106 and 0.460-1.052 in leg and breast met respectively. The Free radical scavenging activity varied significantly and DPPH values of breast meat ranged from 20.70% to 39.09% and in leg meat 23.53% to 43.09% respectively. The sensory acceptability of broiler meat nuggets was decreased with the increase in the level of flaxseeds due to the lipid peroxidation of polyunsaturated fatty acids (PUFA) which generated off flavors and bad odors. Feeding extruded flaxseed to chicken through feed strongly inflated the quality and functional properties, fatty acid contents and reduced the oxidative stability of broiler meat and meat products. The present study concludes that up to 10% of flaxseed meal may be used in broiler diet to enhance the omega 3 fatty acids content in the broiler meat.

## Introduction

Flaxseed is among unique oil seed crops because of its exceptionally high content of α-linolenic acid (ALA), contains 35 to 45% oil, of which 45 to 52% is α-linolenic aci [1]. Flaxseed *(Linum Usitatissimum L.)* is *also* one of the oldest domesticated crop that is under cultivation in Europe and Asia since dating back thousands of years (3000–5000 BC). Approximately more than two hundred species of flaxseed have been recognized [2]. Flaxseed is grown as oil crop, as fiber crop and oil is extracted from oilseed varieties. A large proportion of flaxseed comprises nutritional components such as oil, soluble fiber, protein, lignans, vitamins and minerals [3].

Nutritionists during last two decades are actually clear about selected foods that play an important role in maintaining physical and mental health status of consumers. Beyond meeting nutrition needs, it is generally recognized that dietary factors are considered to modulate detrimental development of some chronic diseases. With increased consumption of highly saturated fat foods, it seems feasible that modern diets do not meet healthy eating guidelines and deficient in certain long chain omega-3 fatty acids. It is widely accepted that inadequate intakes of omega-3 polyunsaturated fatty acids (PUFA), principally alpha-linolenic acid (ALA) and docosahexaenoic acid (DHA), adversely affect cardiovascular function [4]. Flaxseed is rich source of omega-3 polyunsaturated fatty acids and it is helpful in prevention of cardiovascular diseases and cancer. The flaxseed contains all essential amino acid of the protein. It is an excellent source of fiber, lecithin, vitamins and minerals. Moreover, flaxseed is of fastidious significance for its role in lowering the risk of breast and colon cancers [3]. A large proportion of flaxseed comprises nutritional valuable components such as protein (200-240 g/kg), dietary fiber (250–280 g/kg) and flax oil (350-450 g/kg).

The health benefits are related with the ingestion of polyunsaturated fatty acids (PUFA) and dietary fiber. The flaxseed oil contains almost 50% essential fatty acid like alpha-linolenic acid of the total fatty acids that play a significant role in reducing the risk of coronary heart diseases [5]. However, the drawbacks with the dietary inclusion of flaxseed are the presence of anti-nutritinal factors, which may limits its utilization in poultry diets. Mucilage coming from hull, linatin dipeptide (vitmine B6 antagonistic), cyanogenic glycosides, trypsin inhibitor and phytic acid are the most important antinutritional factors found in flaxseed [6].

Alpha-linolenic acid (ALA) and linoleic acid (LA) are the essential fatty acids among the polyunsaturated fatty acids. Human body cannot synthesize alpha-linolenic acid linoleic acid so they should be present in the diet. LA and LNA are metabolized to long chain PUFA in mammalian cells. The omega-6 fatty acid LA is converted to γ-LNA and diomo-γ-LNA by desaturase and elongase enzymes to produce arachidonic acid (AA). AA is further metabolized to eicosanoids or docosapentaenoic acid (DPA). LNA which is omega-3 fatty acid is converted to stearidonic acid and eicosatetraenoic acid to synthesize eicosapentaenoic acid (EPA) by the same enzymes used in synthesis of AA. Eicosapentaenoic acid is then metabolized to docosahexaenoic acid (DHA) or eicosanoid [7].

The limiting consumption of flaxseed is due to presence of anti-nutritional factors such as cyanogenic compounds and tannin which negatively influence the health and well-beings of the masses. The cyanogenic glycosides (CG) are amino acids derived from flaxseed constituents which release a potent cell respiration inhibitor; toxic hydrogen cyanide (HCN) via hydrolysis. The presence of these compounds restricts flaxseed applications in feed and foodstuff by producing bitterness in final products [8]. Presence of phytic acid in the diet reduces micronutrient bioavailability and metabolizeable energy which ultimate lead towards poor growth of poultry birds. Anti-vitamin B6 compound named as ''linatine'' reduces dry matter and produces vitamin B6 deficiency. Omega-3 enriched meat has several health benefits. It plays an important role in prevention or treatment of cardiovascular disease, hypertension, arthrosclerosis, cancer neurological disorders and inflammatory disease. The increased intake of omega-3 fatty acids decreases serum cholesterol which beneficially affects blood pressure, skin diseases, thrombosis atherosclerosis and diabetes, arterial compliance and hyperlipidemia response [9].

Extrusion cooking is highly adaptable and focused form of processing in which food or feedstuff is enforced to flow under controlled conditions of heating and shearing through a terminal die. Extrusion processing of feed ingredients has become very popular during the last two decades. The principal features of extrusion cooking include flexible product characteristics, high energy efficiency, less space required for operation, new feed products formulations, automated control system with high productivity and product quality with no effluent showing environmental-friendly technology to be used. Optimized results for temperature (146.0°C), feeding rate (32.7 kgh−1), screw speed (152.5rpm), moisture content (12.5%) and the hydrocyanic acid removal rate (93.23%) were founded during the extrusion detoxification technique on flaxseed via stepwise non-linear regression analysis and response surface method. It has been demonstrated that maximum removal rate of mucilage (60.3%) from flaxseed meal can be achieved at different co-rotating twin-screw extruder die temperatures (80-160°C), screw speeds (300-900rpm) and initial moisture contents ranged as 18.8-35.1% [10]. There exist a relationship between the stability of the flaxseed derived protein and antifungal activity with temperature variable (50-90°C) using response surface methodology [11]. Regression analyses recommended the significant negative consequences on the residual antifungal activity against all test fungi using treatment variable. The protein content exits in flaxseed by means of response methodology with 5 central points and 4 axial points for independent variables [12]. Extrusion cooking improved the degradation of flaxseed mucilage and negatively influences the compact fiber structure flow behavior index with the addition of initial moisture content, high temperature and decreased screw speed [13].

Incorporation of n-3 fatty acids from flaxseed into eggs and meat is an efficient process tool to increase the polyunsaturated fatty acids content of meat and eggs. The chicken is able to digest flaxseed in the digestive tract, absorb nutrients from flaxseed in the small intestine, and then further metabolize some of the alpha-linolenic acid (ALA; C18:3n-3), elongating and desaturating the ALA to eicosapentaenoic acid (EPA; C20:5n-3) and docosahexaenoic acid (DHA; C22:6n-3) for deposition in lipid stores such as egg yolk, and phospholipids in meat products. Flaxseed is a valuable and natural source of n-3 fatty acids for the poultry producer wanting to market an n-3 polyunsaturated fatty acid (PUFA) enriched poultry product. 300 mg of omega-3fatty acids per 100 g of breast meat can be achieved in 26.2 days at 10% ground flaxseed meal [14]. Keeping in view the breakthrough results in flaxseed or flaxseed meal studies, the present study was carried out to compare the effects of extruded flaxseed meal supplementation on broiler growth performance, oxidative stability, ALA, polyunsaturated fatty acids content, quality attributes of broiler meat and sensory acceptability of meat products.

## Materials and methods

### Composition of diet

The composition of the control feed was 300 g of corn, 200.0 g of soya been meal of 44% protein, 140.0 g of Sorghum, 100.0 g of sunflower meal, 60g of barley, 50 g of rye, 50.0 g of beef tallow, 20 g of dicalcium phosphate, 2 g of sodium bicarbonate, 10 g of salt and 10.0 g of Sepiolite per kg of feed. The analysis of feed revealed that metabolizable energy of the control feed was 2850 kcal/kg, crude protein (16.0%), crude fat (4.1%) fiber (5.1%) and ash content was (7.4%). Additionally flaxseed was supplemented in the feed at a level of 5, 10 and 15% as mentioned in the treatment plan (Table 1).

### Extrusion of flaxseed

Hammered milled flaxseed was extruded using the single screw extruder, Extru-tech E325 (Extru-tech, Sabetha, Kansas). Designed single screw extruder was consisting of feed delivery system, heating arrangement, preconditioning system, power transmission system and extruder barrel. Optimum pre-conditioning was carried out by the addition of steam in the pre-conditioner part of extruder prior extrusion heating of flaxseed. The conditions for extrusion of flaxseed were extruder final head temperature (80-120°C), extruder barrel speed (80-120rpm) and feeder speed (12-16rpm) as described by [15]. The extruded flaxseed was then added to broiler diet.

### Experimental birds management

One day old (120) broiler chicks of Hubbard strain were purchased from local market. The chicks were weighed individually and then randomly divided in to 12 experimental units of 10 birds each. These units were further allotted to 4 treatments in such a way that each treatment has 3 replicates. The birds were kept in a student reserved research room having 31 pens of 12 sq feet capacity each. The room and the pens were cleaned thoroughly, white washed and then disinfected with formalin with a ratio 1:12 inside and outside the pen before the start of experiment. A layer of saw dust was used as litter in each pen that was stirred regularly during the experiment to keep it in dry condition. The temperature of the experimental room was maintained at 95°F during the first week. It was then lowered by 5°F till it reached 75 F° ±2. Twenty four hour light and proper ventilation was provided in the experimental room throughout the experimental period. Feed along with fresh and clean water was given *ad-libitum*. The experimental birds were reared up to six weeks. First two weeks, the chicks were fed on the control feed and after the four weeks the feed was supplemented with extruded flaxseed.

### Sample collection

After the completion of the 6 weeks, the birds were slaughtered according to the Halal ethical guidelines of slaughtering set by the university and white (breast) and red meat (leg) were separated and packaged in polythene zip lock bags and stored at −18°C for further analysis.

**Table 1:** Treatment plan of experimental birds

**Treatments**	**Treatments combination**
T_0_	Control
T_1_	Control + 5% extruded flaxseed
T_2_	Control + 10% extruded flaxseed
T_3_	Control + 15% extruded flaxseed

### Analysis of broiler meat

#### Sample preparation for analysis

5 gram meat sample was taken in 50 ml polypropylene tube having a cap and sample homogenized by using phosphate buffer and glycerol (20%) pH (7.4) with the help of homogenizer. During the process of homogenization, tubes were placed in ice cold water to dissipate the heat. Filtration process was done to remove connective tissues from sample by using muslin cloth.

### Enzyme assay

The superoxide dismutase (SOD) activity was determined by measuring the ability of enzyme to inhibit cytochrome ‘c’ oxidation [16]. The catalase (CAT) activity was measured by observing decomposition of hydrogen peroxide [17] and one international unit (IU) was equivalent to one mmole H_2_O_2_ consumed/min/mg protein. The Glutathione peroxidase (GPX) activity was estimated by using the method of [18] and activity was measured as nmol of NADPH/min/mg Protein. The Glutathione reductase (GR) activity was assayed by following the reduction of glutathione [19].

### 1, 1-diphenyl-2-picrylhdrazyl (DPPH) scavenging activity

The free radical scavenging activity of leg and breast meat was determined by following modified method of [20]. Meat sample having protein concentration (1mg/1mL) took 100 μL fraction volumes in a test tube. Freshly prepared 0.0012M DPPH (1, 1-diphenyl-2- picrylhydrazyl) in methanol solution was added 2 mL in tubes containing 100 μL volume of sample. The reaction was incubating for 30 minute at room temperature in dark place. Absorbance of resultant mixture was recorded at 517 nm against blank and control using UV/Vis. spectrophotometer.

### Thiobarbituric acid reactive substances (TBARS) assay

The oxidative stability of omega enriched broiler meat was determined by using TBARS assay [21]. Ferrous sulphate and hydrogen peroxides were added to start the peroxidative reaction. The solution was placed in water bath at 37°C. 1 ml sample was taken from reaction mixture in glass tube and 2 ml TBARS/TCA solution was added. Test tubes were placed in water bath at 90°C and kept for 30 minutes and then cooled. Then centrifugation was done and the absorbance was recorded at 532 nm and lipid peroxidaton was calculated by using the formula

$$\mathrm{n-Mole}\;\mathrm{of}\;\mathrm{malondialdehyde}=\frac{\left(\mathrm{Sample}\;\mathrm{absorbance-blank}\right)\;\times \;\mathrm{Total}\;\mathrm{sample}\;\mathrm{volume}}{0.000156\times 1000\left(\mathrm{per}\;\mathrm{mL}\right)}$$

### Fatty acids profile of broiler meat

The polyunsaturated fatty acid content of omega three enriched broiler meat was determined by using gas liquid chromatography [22]. Lipids from meat samples were extracted using a non-polar solvent, n-hexane (GC-grade). The fatty acids profile of extracted lipids were prepared and analyzed according to the AOCS (1998) Method No. Ce 1f-96. 50 μl oil sample was methyated in the presence of 4 mL KOH (1 M) at room temperature for one hour in order to produce fatty acids methyl esters. The resultant methyl esters were extracted with GC grade n-hexane for immediately analyzing by Gas Chromatograph (Agilent Technologies, 6890N) equipped with an auto sampler, flame-ionization detector (FID) and fused capillary column (Silica 30m × 0.25 film thickness) apparatus. Samples (1 μL) were injected with Nitrogen (3.5 mL/min) as a carrier gas onto the column, which was programmed for operating conditions such as column oven temperature 220°C for 7.5 minutes, split ratio (50%) with injector and detector temperatures (260°C). Peak areas and total fatty acids profile percentages were calculated for each sample by retention time using Agilent Chem. Station software. The standards of fatty acids methyl esters purchased from Sigma-Aldrich were also run under the same conditions for comparison with experimental samples.

### Product development

#### Preparation of nuggets

The nuggets of omega 3 enriched broiler meat were prepared by the method described by [23]. The basic recipe of the nuggets is as Chicken boneless 500 g, Egg 1, oil as required for frying, Black pepper 12 g, Garlic paste 10 g, Onion 100 g, Plain flour 120 g, Bread crumbs 70 g, salt 20 g. Hand deboned chicken breasts and leg meat was separated and utilized for the development of the nuggets. Breast and leg meat was minced separately with respective additives as mentioned in the recipe of nuggets. All ingredients were mixed and then uniformly blended to obtain a uniform mixture. Then sheeting was done and nuggets were shaped into 30 mm diameter. After nuggets were prepared, they were dipped in plain flour and bread crumbs separately. The frying of the nuggets was done in canola oil at 180°C till golden brown color was appeared.

### Physico-chemical analysis of nuggets

The pH of Nuggets was measured by using pH meter by following the method as described by [24]. The colour of nuggets was measured by using a hand held tristimulus colorimeter (Color Test Meter II) at regular storage intervals (0,10,20,30 days) as described by [25]. The water activity of Nuggets was determined by using an electronic Hygropalm water activity meter (Model Aw-Win, Rotronic, equipped with a Karl-Fast probe) as described by [26] The textural characteristics of sausages were analyzed at different storage intervals by means of texture analyzer (Mod. TA-XT2, Stable Microsystems, surrey, UK) Texture was determined with the help of texture analyzer (model TA_XT Plus, Stable Microsystems, Surrey, UK) as described by [27].

### Sensory evaluation of nuggets

The sensory evaluation of fried nuggets was carried out for the different attributes like color, appearance, taste, texture and off flavor production by using nine point hedonic scale after 0, 10, 20 and 30 days, by using the method as described [28] by trained panelists as the method described.

### Statistical analysis

The data obtained for each parameter was subjected to statistical analysis to determine the level of significance by using the software package (Statistic 8.1) according to the method described [29]. The Duncan’s multiple range (DMR) test was used to estimate the level of significance that existed between the mean values.

## Results and discussion

### Growth parameters

The results in (Table 2) showed that there was a highly significant effect of different treatments of extruded flaxseed on weight gain of the broiler birds. T_0_ control treatment having no extruded flaxseed gained highest weight 960.61 g followed by the T1 containing 5% extruded flaxseed having weight 931.56 g and T_2_ containing 10% extruded flaxseed 891.61and T_3_ containing maximum extruded flaxseed 15% gained lowest weight 850.06g. The weight gain performance of broilers was affected negatively by the increased level of extruded flaxseed. The results of my study match with the finding of [30]. They observed that the flaxseed depressed the body weight gain in the broiler birds. The depression in bird performance appears to be due to poor energy availability [31]. The feed intake of broiler birds was significantly affected by different treatments of extruded flaxseed. Feed intake increased with the increase in time period and decreased with the increase in extruded flaxseed level. T_0_ contains no flaxseed showed highest feed intake 651.44g and the T_3_ containing maximum level of extruded flaxseed showed the lowest feed intake 626.89g. T_1_ (640.8g) and T_2_ (633.6) the feed intake was higher than the T_3_ but lower than the T_0_. The results of my study are in agreement with the finding of [32] who stated that the feed intake decreased with high level of flaxseed in bird’s feed. The feed intake may be reduced due to the high fat contents in the extruded flaxseed supplemented diet. The other factor that is important in lowering the feed intake is the antinutritional factor present in flaxseed even after the extrusion of flaxseed. The feed conversion ratio is the ratio between weight gain and feed intake of birds and represents the efficiency of birds to convert feed into body weight. The results for feed conversion ratio showed the significant effect of different treatments of extruded flaxseed on feed conversion ratio of broilers. The feed conversion ratio increased with the increase in the extruded flaxseed level. T_3_ showed the highest FCR (2.007) followed by T_2_ (1.93), T_1_ (1.88) and minimum was observed in T_0_ as (1.845). The highest FCR value in high extruded flaxseed treatment is due to the less weight gain in the broiler birds. The results of my study are in close collaboration with the [33] who stated that the bird fed with high flaxseed levels have high FCR values which may be due to the low digestibility and high viscosity of jejunal digesta.

**Table 2:** Body weight gain (g/week)) and feed conversion ratio of the broiler birds fed on extruded flaxseed on weekly basis

**Study weeks**	**Dietary treatment**			
	**Control (C)**	**(C)**	**(C)**	**(C)**
		**+**	**+**	**+**
		**5% EFM**	**10% EFM**	**15% EFM**
**Wk-1**	94.0±3 (BWG) 1.71±0.04 (FCR)	94.3±2(BWG) 1.69±0.09(FCR)	93.7±4(BWG) 1.68±0.03(FCR)	92.7±5(BWG) 1.73±0.10(FCR)
**Wk-2**	332±11(BWG) 1.53±0.07(FCR)	315±14(BWG) 1.65±0.09(FCR)	302±21(BWG) 1.75±0.15(FCR)	289±27(BWG) 1.86±0.20(FCR)
**Wk-3**	644±20(BWG) 1.86±0.11(FCR)	631±20(BWG) 1.79±0.08(FCR)	600±39(BWG) 1.86±0.07(FCR)	560±14(BWG) 2.03±0.19(FCR)
**Wk-4**	1054±33(BWG) 1.83±0.15(FCR)	1018±30(BWG) 1.91±0.14(FCR)	977±45(BWG) 1.94±0.15(FCR)	926±38(BWG) 1.98±0.20(FCR)
**Wk-5**	1582±47(BWG) 1.81±0.24(FCR)	1542±41(BWG) 1.82±0.28(FCR)	1470±35(BWG) 1.90±0.24(FCR)	1408±33(BWG) 1.93±0.25(FCR)
**Wk-6**	2058±54(BWG) 2.31±0.30(FCR)	1989±45(BWG) 2.38±0.32(FCR)	1907±48(BWG) 2.41±0.29(FCR)	1824±42(BWG) 2.48±0.31(FCR)
**Means±SD**	960.61±28A(BWG) 1.84±0.15B(FCR)	931.56±25B(BWG) 1.88±0.16B(FCR)	891.61±32C(BWG) 1.93±0.15AB(FCR)	850.06±26D(BWG) 2.00±0.20A(FCR)

### Enzyme assay of broiler blood

All living organisms have natural defence system against the free radicals produced by the consumption of oxygen. This defence system contains different types of antioxidant enzyme. Most important enzyme are superoxide dismutase (SOD), catalase (CAT), glutathione peroxidise (GSH-Px) and glutathione reductase (GR). The results (Table 3) revealed significant effect of different treatments of extruded flaxseed on the different enzymes assay in the plasma of the broiler blood. The superoxide dismutase activity ranged from 2.80 to 3.22 U/ml. T_3_ showed the highest 3.22 U/ml superoxide dismutase activity while T_1_ and T_2_ were also affected significantly as compared to control treatment. The catalase activity of blood ranged from 36.38 to 45.42 (mmol of H_2_O_2_ decomposed/min/mg). Catalase activity increases toward the treatment T_3_ (45.42) which have maximum level of extruded flaxseed. T_1_ and T_2_ also affected the catalase activity (38.75 and 42.46) as compared to the control treatment (36.38). The catalase activity is related to the SOD activity because SOD convert superoxide radicals to hydrogen peroxide. More production of the superoxide radicals more will be the activity of SOD and hence more the activity of catalase. The values for GSH-Px ranged from 2.33 to 3.23. The highest glutathione peroxidase activity was observed in T_3_ (3.23) and the minimum in T_0_ (2.3). The higher GSH-Px values higher will be the oxidation. Lipid oxidation and cholesterol oxidation were also positively correlated with the GSH-Px activity. GSH-Px activity can be used as an indicator of the meat oxidative stability. The higher GSH-Px activities observed for the different treatments of extruded flaxseed may be due to the higher content of PUFA of diet. The results of my study are in agreement with [34]. Glutathione reductase (GR) activity in different treatment ranged from 3.93 to 4.33 u/g of protein. The activity of the GR increased towards the treatment containing high amount of extruded flaxseed.T_3_ showed maximum activity 4.33 u/g. GR activity is induced by the by the unsaturated and polyunsaturated fats as compared to the saturated fats. The increase in activity of GR in red blood cells is a positive feedback mechanism in response to rising lipid peroxidation [35].

**Table 3:** Antioxidant enzymes of blood serum of broiler birds fed on different levels of extruded flaxseed through feed

**Treatments**	**SOD (50% Pyrogyllol auto-oxidation/min/mg)**	**CAT (mmol of H**_**2**_**O**_**2**_**decomposed/min/mg)**	**GSH-Px activity (nmol NADPH/min/mg Protein)**	**Glutathione reductase (u/g protein)**
**T**_**0**_	2.80±0.06d	36.38±0.88d	2.33±0.06c	3.93±0.06c
**T**_**1**_	2.99±0.01c	38.75±1.04c	2.45±0.05c	4.07±0.05b
**T**_**2**_	3.09±0.03b	42.46±0.82b	2.93±0.04b	4.26±0.04a
**T**_**3**_	3.22±0.03a	45.42±0.88a	3.23±0.09a	4.33±0.06a

Values are mean of three replicates ± SD. Means followed by different letters are significant different (p<0.05).

### Oxidative stability of broiler meat

#### Thiobarbituric acid reactive substances (TBARS) assay

Lipid peroxidation is the most important factor of quality deterioration in meat. The thiobarbuturic acid assay is a practical method for the determination of food lipid peroxidation. The results (Table 4) revealed a highly significant effect of different treatments of extruded flaxseed on TBARS value of leg and breast muscles of broilers. Leg meat TBARS values ranged from 0.850-2.106. T_0_ control treatment having no extruded flaxseed showed the minimum TBARS value 0.850 and T_3_ containing highest extruded flaxseed level (15%) showed the maximum TBARS value 2.106. T_1_ and T_2_ have TBARS values 1.289 and 1.856 respectively. The increasing trend in TBARS values was observed as the level of extruded flaxseed increased. High oxidative deterioration in broiler meat is due to its high concentration of polyunsaturated fatty acids [36]. The results of my study are similar with the findings of [14] who observed that the TBARS values were higher in higher level of flaxseed feeding to broiler birds. Breast meat also exhibited the same pattern as leg meat. T_3_ showed the highest TBARS value1.052 as compared to T_1_ and T_2_ which were 0.588 and 0.796 respectively. TBARS values were higher in all treatments of extruded flaxseed as compare to the control treatment. Results of my study are agreed with [37]. The TBARS values were less in breast meat as equate with leg meat and it may be due to the less amount of fat contents in the breast meat. The results (Table 4) revealed that all the treatments of extruded flaxseed significantly affected the free radical scavenging activity. The free radical scavenging activity of leg meat ranged from 22.58 to 43.94% while in breast meat ranged from 20.70 to 39.09. The reason might be that high flaxseed feeding in diet results in high free radical scavenging activity and lignans play vital role in the raising the freed radial scavenging activity.

**Table 4:** Total Lipids, TBARS assay, Free radical scavenging activity of leg and breast meat of broilers fed on extruded flaxseed meal supplemented diet

**Treatments**	**Meat type**					
	**Breast meat**			**Leg meat**		
	**Total lipids (%)**	**TBARS assay (nmol of malondialdehyde/mg of meat)**	**Free radical scavenging activity (%)**	**Total lipids (%)**	**TBA assay (nmol of malondialdehyde/mg of meat)**	**Free radical scavenging activity (%)**
**T**_**0**_	7.88±0.4b	0.460±0.03d	19.87±1.1c	9.97±0.5d	0.850±0.01d	23.53±1.1d
**T**_**1**_	8.10±0.4b	0.588±0.01c	25.98±3.9b	12.80±0.5c	1.289±0.03c	28.30±1.8c
**T**_**2**_	8.63±.0.4a	0.796±0.01b	33.13±2.6a	15.10±0.8b	1.856±0.03b	35.25±2.6b
**T**_**3**_	8.96±0.3a	1.052±0.01a	38.27±2.6a	18.92±0.7a	2.106±0.02a	43.09±1.1a

Values are mean of three replicates ± SD. Means followed by different letters are significant different (p<0.05).

### Fatty acid analysis of broiler meat

The fatty acid profile (mg/100 g of meat) corresponding to leg and breast meat is shown in Table (5) and it revealed that palmitic acid contents in leg meat ranged from 58.64 to 62.73 (mg/100 g). It is cleat form results that the maximum value of palmitic acid (62.73) was found in T_3_ followed by T_2_ (61.77), T_0_ (58.64) and T_1_ (57.97) While the breast meat contained 55.87 to 6.91 (mg/100 g) palmitic acid. The results regarding stearic acid showed that there was maximum stearic acid present in T_3_ (44.68) and minimum was observed in T_0_ (33.65) in leg meat. The stearic acid contents in breast meat ranged from 27.65 to 37.59. The GC analysis pertaining to oleic acid contents of leg meat was presented in Table (4.21). Oleic acid contents ranged from 59.87 to 70.52. Maximum oleic acid contents was present in T_2_ (7.52) followed by T_3_ (68.99). The breast meat oleic acid contents ranged from 56.22 to 66.57. Highest oleic acid contents were found in T_3_ (66.57). Linoleic acid contents in leg meat ranged from 69.12 to75.68. T_3_ showed the highest linoleic contents (75.68) followed by T_1_ (74.31) and T_2_ (73.36). The breast meat linoleic contents ranged from 51.88 to 74.14. Minimum linoleic contents were observed in T_1_ (51.88). Omega-3 fatty acid (linolenic acid) ranged from 3.98 to 9.52. Maximum lilenic acid was present in T_3_ (9.52) followed by T_2_ (8.41), T_1_ (6.10) and T_0_ (3.98). Breast meat contained linolenic acid in the range of 1.96 to 3.24. The linolenic acid contents in breast meat were less as compared to the thigh meat. Thigh meat fatty acid profile was highly enriched by the different concentrations of extruded flaxseed in the diet of birds. The higher addition of linolenic acid in fatty acid profile of leg meat was due to its high capacity of modification in lipid fraction. The results agreed with [Zuidhof *et al.* (2009]). Similar results were reported in rabbits when fed with different levels of flaxseed [38] and in pork [39]. All these authors reported that there was significant increase in polyunsaturated fatty acid contents of fatty acid profile when the flaxseed was given through the diet to the broilers.

**Table 5:** Fatty acid profile of breast and leg meat of broilers fed on extruded flaxseed meal supplemented diet

**Treatments**	**Meat type**							
	**Breast meat**				**Leg meat**			
	**T**_**0**_	**T**_**1**_	**T**_**2**_	**T**_**3**_	**T**_**0**_	**T**_**1**_	**T**_**2**_	**T**_**3**_
**C8**	0.28	0.31	0.27	0.26	0.27	0.27	0.25	0.26
**C10**	0.45	0.47	0.43	0.41	0.41	0.41	0.4	0.39
**C14,0**	0.52	0.63	0.51	0.49	0.57	0.56	0.55	0.54
**C16,0**	55.87	58.76	57.42	60.91	58.64	57.97	61.77	62.73
**C16,1**	1.92	1.94	1.91	1.89	1.98	2.01	1.93	1.91
**C17,0**	0.32	0.33	0.3	0.31	0.2	0.19	0.18	0.17
**C17,1**	0.19	0.2	0.18	0.17	0.07	0.06	0.07	0.05
**C,18,0**	27.65	25.72	33.45	37.59	33.65	40.61	39.52	44.68
**C18,1**	56.22	60.62	59.12	66.57	33.29	35.29	33.05	32.17
**C18,2**	54.81	51.88	68.74	74.14	69.12	74.31	73.36	75.68
**C18,3**	1.96	2.76	3.02	3.24	3.98	6.1	8.41	9.52
**C20,4**	5.88	5.87	5.87	5.8	5.48	5.5	5.44	5.26
**C22,0**	0	0.09	0	0	0	0.1	0	0
**C22,1**	0	0.13	0	0	0	0.15	0	0
**C22,5**	0.09	0.07	0.08	0.08	0.11	0.1	0.09	0.1
**C22,6**	12.22	17.66	23.11	27.65	11.55	19.23	23.12	26.67
**SFA**	57.44	86.31	92.38	99.97	93.74	100.11	102.67	108.77
**MUFA**	58.33	62.89	61.21	68.63	35.34	37.51	35.05	34.13
**PUFA**	74.96	78.37	100.82	110.91	90.24	105.39	110.42	117.23
**US**	133.29	141.26	162.03	179.54	125.58	142.9	145.47	151.36
**Total**	190.73	227.57	254.41	279.51	219.32	243.01	248.14	260.13

SFA= Saturated Fatty acids, MUFA=Mono Unsaturated Fatty acids, PUFA= Poly Unsaturated Fatty acids US=Unsaturated Fatty acids.

### Physico-chemical analysis of nuggets

pH of the nuggets is an important factor in the determination of the shelf life and storage stability of the nuggets. The results (Table 6) revealed the treatments have no significant effect on the pH of the nuggets while the storage affected the pH significantly at freezing storage conditions. However, the interactive effect of storage days and different treatments of flaxseed was also non significant. At day 0, T_0_ had pH value 6.64 which reduced to 6.59 at day 30. T_1_ showed the same pattern at 0 day pH value of nuggets was 6.66 which decreased to 6.61 at day 10 and 6.58 at day 20 and ultimately 6.50 at day 30. T_2_ also showed the decrease in pH during storage. The pH was decreased from 6.77 to 6.61 from 0 to 30 days.T_3_ followed the same pattern as T_0_, T_1_and T_2_ the pH 6.71 at 0 days decreased up to 6.54 at day 30. The results of present study are in close collaboration with [40] who stated that pH value of omega enriched frankfurters decreased with the advancement of storage period.

**Table 6:** pH, water activity, color and texture values of nuggets of leg and breast meat of broilers fed on extruded flaxseed meal supplemented diet

**Treatment**	**Breast meat nuggets**					**Leg meat nuggets**				
**pH**	**0 day**	**10 day**	**20 day**	**30 day**	**Mean**	**0 day**	**10 day**	**20 day**	**30 day**	**Mean**
**T**_**0**_	6.69±0.03	6.63±0.02	6.60±0.02	6.56±0.07	6.62±0.05ab	6.64±0.05	6.64± 0.06	6.63±0.02	6.59±0.04	6.6267±0.02 a
**T**_**1**_	6.64±0.07	6.60±0.05	6.58±0.07	6.53±0.05	6.58± 0.04b	6.66±0.06	6.61±0.03	6.58±0.07	6.50±0.03	6.5883±0.06ab
**T**_**2**_	6.77±0.11	6.64±0.05	6.62±0.02	6.61±0.04	6.66±0.07a	6.64±0.07	6.62±0.02	6.55±.0.09	6.50±0.02	6.5783±0.06 b
**T**_**3**_	6.71±0.11	6.66±0.07	6.57±0.07	6.54±0.03	6.62±0.07ab	6.65±0.04	6.59±0.05	6.58±0.06	6.49±0.03	6.5750±0.06 b
**Mean**	6.70±0.05a	6.63±0.02b	6.59±0.02bc	6.56±0.03c		6.65±0.01a	6.62±0.02ab	6.59±0.03b	6.52±0.c	
**Water activity**										
**T**_**0**_	0.683±0.005	0.667±0.002	0.663±0.005	0.655±0.004	0.6669±0.01a	0.671±0.003	0.670±0.004	0.660±0.004	0.654±0.002	0.6638±0.008a
**T**_**1**_	0.678±0.004	0.672±0.003	0.670±0.005	0.647±0.005	0.6668±0.01a	0.664±0.004	0.660±0.005	0.658±0.004	0.648±0.002	0.6575±0.006b
**T**_**2**_	0.673±0.005	0.668±0.005	0.662±0.007	0.659±0.003	0.6657±0.00a	0.659±0.005	0.656±0.004	0.650±0.004	0.646±0.005	0.6528±0.005c
**T**_**3**_	0.676±0.004	0.661±0.004	0.655±0.004	0.643±0.003	0.6589±0.013b	0.656±0.03	0.655±0.003	0.649±0.004	0.644±0.006	0.6510±0.005c
**Mean**	0.6777±0.004A	0.6672±0.00B	0.6627±0.00C	0.6508±0.0D		0.6626±0.0A	0.6602±0.00A	0.6541±0.5B	0.6482±0.04C	
**Color**										
**T**_**0**_	115±4	111±4	110±2	102±4	109.75±5a	125±3	120±3	111±3	106±3	115.83± 9a
**T**_**1**_	110±3	103±3	102±2	99±1	103.50±5b	122±2	119±4	110±3	100±2	112.67±10b
**T**_**2**_	111±5	106±4	98±3	95±3	102.58±7b	121±3	117±3	106±3	100±3	110.92±10bc
**T**_**3**_	107±3	105±2	92±3	90±2	98.67±9 c	119±1	118±3	104±2	99±1	109.92±10c
**Mean**	110.92±3 A	106.33±3B	100.67±8C	96.58±5 D		121.83±3 A	118.42±1 B	107.50± 3 C	101.58± 3D	
**Texture**										
**T**_**0**_	985.47±4.90	986.83±4.31	988.53±5.68	991.10±4.09	987.98±2.42A	969.3±3.4	973.9±3.1	976.7±4.0	978.3±3.2	974.57±3.9AB
**T**_**1**_	984.33±5.06	984.83±5.41	985.60±4.90	991.37±3.00	986.53±3.26A	974.8±3.9	976.6±4.6	977.5±4.2	979.1±3.8	977.00±1.8A
**T**_**2**_	979.37±4.16	981.97±3.0	986.40±5.47	992.90±4.37	985.16±5.9 A	971.6±3.6	976.0±4.8	978.4±4.3	980.0±4.4	976.49±3.6AB
**T**_**3**_	983.73±4.92	984.53±4.4	986.33±5.65	994.33±3.74	987.23±4.85A	968.9±3.6	970.0±3.0	974.7±3.3	979.6±5.0	973.30±4.9B

Values are mean of three replicates ± SD. Means followed by different letters are significant different (p<0.05).

Water activity (a_w_) is the most important property of water in food system. Scott (1957) reported that there is an optimum water activity level below that the micro organism cannot grow well. The results showed that the different treatments of extruded flaxseed have significant effect on water activity of leg meat nuggets. Water activity of leg meat nuggets ranged from 0.683-0.676. During storage the water activity of leg mat nuggets decreased as the time period increased. At 0 day all the treatments have higher water activity as compared to the 10, 20 and 30 days. The lowest water activity was observed at 30 in all treatments. Water activity of breast meat nuggets ranged from 0.671-0.656. During storage the breast meat nuggets water activity behavior was same as that of leg mat nuggets. The water activity decreased with the passage of time during storage and minimum water activity was observed at day 30. The results of my study are in contrast with the findings of [41] who observed no change in water activity in omega enriched sausage during storage. This variation in results may be due to the different level of enrichment or different storage conditions.

Food appearance is one of the major factors that determine the consumer acceptance and ultimately the sale of product. Food quality and shelf life is limited by the oxidation of polyunsaturated fatty acids and bacterial contamination. The results revealed a highly significant effect of different treatment of extruded flaxseed and storage days on color of the broiler meat nuggets. However the interactive effect of treatments and storage days was significant. The color of leg meat nuggets ranged from 115–107. During storage the values for the color decreased with the passage of time. The decrease in value indicated that the nuggets become darker during storage. In T_3_ minimum value 90 was observed at day 30. The color values decreased in all the treatments of extruded flaxseed during storage. The color values for breast meat nuggets fanged from 125–119. During storage the breast meat nuggets showed the same behavior as the leg meat nuggets. The color values tend to decrease with the storage days increase. The values for color decreased from 0 day to 30 days to the lowest level in all the treatments of extruded flaxseed. The results of my study are in close collaboration with the finding of [42], who studied the color characteristics of omega enriched sausages and found that the color become dark with the passage of time.

Texture is an important tool to check the acceptability of meat products during its storage period. The texture values of leg meat nuggets ranged from 969.3-974.8. During storage the texture values increased with the increase in storage days. The texture value in all the treatments ranged from 969.3 to 974.8 at 0 day which tend to increase 978.3-980.0 at day 30. During storage the breast meat nuggets followed the same pattern as leg meat nuggets. The texture values tend to increase with the storage days. At 0 days the texture values for all the treatments ranged 979.37-985.47 which increased to 991.10-994.33 at day 30. The increase in hardness may be associated with the decrease in water activity of the nuggets with the advancement of storage period. The results of present study are in close collaboration with [43] who stated that the texture value varied significantly with the advancement of storage period.

### Sensory evaluation of nuggets

The development of new food products by modification of ingredients or processing conditions, cost reduction and quality control, often employs sensory evaluation techniques to determine the acceptability of food [44].

Aroma of a product is an important factor involving in the consumer acceptance. It also has contribution in flavor of the product. There was a significant effect of storage and treatments on aroma of nuggets and non-significant results obtained by the interaction of treatments and storage. The results of sensory evaluation presented in table (Table 7) showed that there was a significant decrease in likeness of aroma from 0day to 30 days of storage at freezing temperature. The results pertaining to aroma revealed that T_0_ was preferred by the judges because it gave excellent aroma to nuggets and obtained 7, followed by T_1_ (6.5 points). T_3_ containing maximum level of extruded flaxseed obtained minimum points for aroma by the panel of judges. The aroma tends to less acceptable as the flaxseed level and storage days increased. Minimum score for aroma was observed in T_3_ at day 30 which was 5. The results of my study for aroma are in contrast with [14] who stated that there was no effect of flaxseed levels on the aroma of omega enriched meat products.

**Table 7:** Aroma, flavor, taste and overall acceptability of nuggets of leg and breast meat of broilers fed on Extruded Flaxseed

**Treatment**	**Breast meat nuggets**					**Leg meat nuggets**				
**Aroma**	**0 day**	**10 day**	**20 day**	**30 day**	**Mean**	**0 day**	**10 day**	**20 day**	**30 day**	**Mean**
**T**_**0**_	7±0.50	7±0.40	7±0.0	6±0.50	6.75±0.50 A	6.5±0.40	6.3±0.40	6.2±0.70	6±0.50	6.25±0.50A
**T**_**1**_	7±0.50	7±0.80	6.5±0.5	5.75±0.70	6.5±0.60 AB	6.4±0.60	6.1±0.80	5.8±0.60	5.5±0.80	5.95±0.70B
**T**_**2**_	6.5±0.50	6.5±0.25	6±0.4	5.5±0.25	6± 0.40 BC	6.2±0.50	6.1±0.60	5.7±0.50	5.4±0.30	5.85±0.45B
**T**_**3**_	6±0.40	6±0.25	6±0.0	5±0.50	5.75±0.50 C	6.2±0.80	5.8±0.50	5.6±0.30	5.2±0.50	5.7±0.55 C
**Mean**	6.508±0.5A	6.625±0.50A	6.4±0.50 A	5.50±0.40B		6.25±0.55A	6.05±0.0.60B	5.85±0.50C	5.55±0.45D	
**Flavor**										
**T**_**0**_	7.5±0.40	7±0.75	7±0.00	6±0.50	6.87±0.60 A	7±0.60	6±0.40	6±0.50	5±0.30	6±0.35B
**T**_**1**_	7±0.50	6.5±0.30	6.5±0.50	5.75±0.70	6.45±0.51 B	6.5±0.50	6.5±0.50	6±0.60	6±0.20	6.2±0.45A
**T**_**2**_	7±0.50	6±0.50	6±0.40	5.5±0.25	6.20±0.62 BC	6.5±0.73	6±0.45	5±0.40	5±0.65	5.62±50C
**T**_**3**_	6.5±0.25	6±0.25	6±0.00	5±0.50	5.87±0.62 C	6±0.66	5.5±0.40	5.5±0.70	5±0.40	5.5±0.50C
**Mean**	7± 0.40 A	6.43±0.50 B	6.40±0.50B	5.56±0.43C		6.5±0.60A	6±0.45B	5.75±0.60C	5.25±0.35D	
**Taste**										
**T0**	7±0.50	7±0.40	7±0.00	6±0.50	6.75±0.50 A	7±0.25	7±0.40	6±0.50	5±0.45	6.24±0.40A
**T1**	7±0.50	7±090	6.5±0.50	5.75±0.66	6.58±0.60 A	7±0.45	6±0.50	6±0.60	6±0.50	6.25±0.50A
**T2**	6.5±0.50	6.5±0.25	6±0.40	5.5±0.25	6.14±0.50 B	6±0.50	6±0.35	5±0.50	5±0.25	5.5±0.40C
**T3**	6±0.40	6±0.25	6±0.00	5±0.50	5.7708±0.50B	6±0.60	6±0.40	5.5±0.40	5±0.35	5.75±0.45B
**Mean**	6.64±.0.50A	6.64±0.50A	6.39±0.50A	5.56±0.43B		6.5±0.40A	5.75±0.40B	5.65±0.40C	5.25±00.35D	
**Overall acceptability**										
**T**_**0**_	7.25±0.43	7±0.43	7±0.50	6±0.43	6.81±0.55 A	7±0.45	7±0.50	6±0.40	5±0.45	6.25±0.45B
**T**_**1**_	7±0.50	6.75±0.43	6.5±0.50	5.75±0.50	6.50±0.54 AB	8±0.55	7±0.30	6±0.30	6±0.40	6.75±0.40A
**T**_**2**_	6.5±0.50	6.5±0.25	6.25±0.50	5.5±0.50	6.18±0.47 BC	7±0.60	6±0.20	5±0.50	5±0.60	5.75±0.45C
**T**_**3**_	6.25±0.43	6±0.25	6±0.50	5±0.50	5.81±0.55 C	6±0.40	6±0.40	5±0.60	5±0.50	5.50±0.40D
**Mean**	6.75±0.45 A	6.56±0.43 A	6.43±0.43A	5.56±0.43B		7±0.50A	6.5±0.30B	5.5±0.45C	5.25±0.50D	

Values are mean of three replicates ± SD. Means followed by different letters are significant different (p<0.05).

Flavor is an important parameter in sensory evaluation of a food product; it is the combined perception of smell, taste and mouth feel, making attraction for the consumers. It was obvious from the results that the treatments and storage days had significant effect on the flavor of nuggets; however the interactive effect of treatments and storage days was not significant. The nuggets from the control treatments obtained highest score for the flavor in all the treatments. The flavor values tend to decrease as the level of extruded flaxseed increased. During storage flavor value decreased with the storage days. The minimum value for flavor was observed in T_3_ (5.25) at day 30. The decrease in flavor value is due to the peroxidation of PUFA which results in off flavors and bad odors [45].

Taste is a very important attribute for the acceptance of a food product. The results showed that there was significant effect of treatments and storage days on taste of nuggets. The mean values revealed that T_0_ and T_1_ were much liked by the judges and got highest score, [7] and T_3_ got minimum score [4] at 0 day. During storage the score for taste of nuggets decreased and minimum score was obtained by T_3_[6] at day 30. The decreasing trend in taste of nuggets may be associated with the peroxidation of poly unsaturated fatty acids.

Overall acceptability is another important parameter in sensory evaluation of a product. It determines on the basis of quality scores obtained from aroma, taste, and flavor. The results showed that overall acceptability of nuggets was significantly affected by different treatments while the storage affect was non-significant. T_0_ was highly acceptable by the panelists as got highest score for overall acceptability [7] while T_3_ got minimum score [6] for the overall acceptability at day 30. The results of my study are in agreement with [46] who stated that the overall acceptability decrease with increase in omega enrichment in meat products.

## Conclusion

The present study was designed to investigate the relationship of various quality attributes and storage stability of broiler meat and meat products with different concentrations of extruded flaxseed fed to the broiler birds. The supplementation of extruded flaxseed in the diet of broiler birds resulted decrease in the body weight, feed intake and higher FCR values. T_3_ (15% extruded flaxseed) achieved lowest weight and feed intake. The antioxidant enzyme assay resulted in significant difference among treatments. The chemical analysis of extruded flaxseed fed meat revealed that the fat contents of leg meat greatly increased as compared to breast meat. Maximum fat contents were observed in T_3_ (15% extruded flaxseed). Thiobarbituric acid reactive substances (TBARS) were evaluated in the form of n-moles of malondialdehyde by thiobarbituric acid test which showed the lipid oxidation stability of meat. T_3_ showed the highest amount of n-mole of malondialdehyde which revealed that this group has lowest lipid stability than other groups in this study. Free radical scavenging activity improved in meat by supplementation of higher concentration of extruded flaxseed in diet. The results showed the maximum free radical scavenging activity was found in T3. Fatty acid profile was determined by Gas Chromatography. The results showed higher omega-3 contents in higher level of extruded flaxseed fed meat. Omega-3 fatty acids were deposited according to dose supplemented in the meat. The maximum omega-3 contents were observed in T3 having highest level of extruded flaxseed (15%) than all the other treatments of study. The results from storage study revealed the significant effect of storage on the physicochemical and sensory properties of omega enriched meat nuggets. In the present study it is concluded that as the concentration omega-3 fatty acids increased in broiler meat with the higher level of extruded flaxseed supplementation in diet. Extruded flaxseed supplemented feed increased the total fat contents but decreased the oxidative stability of omega-3 enriched meat. High levels of extruded flaxseed negatively affected the physicochemical and sensory properties of omega-3 enriched nuggets during storage.

The present study concluded that as the concentration of omega-3 fatty acids increased in broiler meat with the higher level of extruded flaxseed supplementation in diet. Extruded flaxseed supplemented feed increased the total fat contents but decreased the oxidative stability of omega-3 enriched meat. High levels of extruded flaxseed negatively affected the physicochemical and sensory properties of omega-3 enriched nuggets during storage.

## Abbreviation

MDA: Malondialdehydes ALA; TBARS: Thiobarbituric Acid Reactive Oxygen Species; HPLC: High-Performance Liquid Chromatography; GC: Gas Chromatography; PUFA: Poly Unsaturated Fatty Acids.

## Competing interests

The authors report no conflicts of interest. The authors alone are responsible for the content and writing of the paper.

## Authors’ contribution

MFHB and MSA carried out the experimental trial of the broilers and also collected data of this research. They also arranged all the data and drafted the manuscript. FMAA provides technical assistance during research of the broiler and also guided in the analysis and statistical design of research trial. MIKC and MSAE also helped to carry out the analytical research work and analysis of the meat and meat products. “It’s also confirmed that all the authors read and approved the final manuscript”.
